# Genetic Interaction Analysis Reveals that Cryptococcus neoformans Utilizes Multiple Acetyl-CoA-Generating Pathways during Infection

**DOI:** 10.1128/mbio.01279-22

**Published:** 2022-06-29

**Authors:** Katy M. Alden, Andrew J. Jezewski, Sarah R. Beattie, David Fox, Damian J. Krysan

**Affiliations:** a Department of Pediatrics Carver College of Medicine, University of Iowa, Iowa City Iowa, USA; b UCB Biosciences, Bainbridge Island, Washington, USA; c Seattle Structural Genomics Center for Infectious Disease (SSGCID), Seattle, Washington, USA; d Beryllium Discovery Corp., Bainbridge Island, Washington, USA; e Microbiology/Immunology, Carver College of Medicine, University of Iowa, Iowa City Iowa, USA; University of British Columbia

**Keywords:** carbon metabolism, *Cryptococcus neoformans*, fungal pathogenesis, acetyl CoA

## Abstract

Cryptococcus neoformans is an important human fungal pathogen for which the external environment is its primary niche. Previous work has shown that two nonessential acetyl-CoA metabolism enzymes, ATP-citrate lyase (*ACL1*) and acetyl-CoA synthetase (*ACS1*), play important roles in C. neoformans infection. Here, we took a genetic interaction approach to studying the interplay between these two enzymes along with an enzyme initially called *ACS2* but which we have found is an acetoacetyl-CoA synthetase; we have renamed the gene 2-**k**eto**b**utyryl **C**oA synthetase 1 (*KBC1*) based on its biochemical activity and the systematic name of its substrate. *ACL1* and *ACS1* represent a synthetic lethal pair of genes based on our genetic interaction studies. Double mutants of *KBC1* with either *ACS1* or *ACL1* do not have significant synthetic phenotypes *in vitro*, although we find that deletion of any one of these enzymes reduces fitness within macrophages. Importantly, the *acs1*Δ *kbc1*Δ double mutant has significantly reduced fitness in the CNS relative to either single mutant as well as WT (~2 log_10_ CFU reduction in fungal burden), indicating the important role these enzymes play during infection. The expression of both *ACS1* and *KBC1* is increased *in vivo* relative to *in vitro* conditions. The *acs1*Δ mutant is hypersusceptible to fluconazole *in vivo* despite its minimal *in vitro* phenotypes. These data not only provide insights into the *in vivo* mechanism of action for a new class of antifungal Acs inhibitors but also into metabolic adaptations of C. neoformans to the host environment.

## INTRODUCTION

Cryptococcus neoformans is an environmental yeast and an accidental human pathogen (1). As a causative agent of Cryptococcosis, it is a major cause of disease in immunocompromised patients. Globally, HIV/AIDS remains the dominant risk factor associated with the development of cryptococcal meningoencephalitis, the primary disease manifestation (2). Understanding the underlying mechanisms that allow this environmental yeast to become a pathogen has been a topic of intense interest to the medical mycology field (3). The majority of these studies have focused on the factors or characteristics that appear to be required for virulence: the so-called Big Three of host temperature tolerance, melanin formation and capsule production (4). Based on large-scale, pheno- and genotyping of diverse clinical and environmental isolates, the expression of these virulence traits is, indeed, strongly associated with disease in humans (5). Despite this correlation, significant variation in virulence toward human patients and in mammalian models of cryptococcosis is observed within highly related isolates that express all three of the canonical virulence traits (6). Accordingly, it is now well-recognized that additional infection-related traits remain to be discovered and characterized (7). Recently, we and others have become interested in the hypothesis that a deeper understanding of this variation may emerge by exploring the traits and processes required for C. neoformans to transition from an environmental niche to the host (8).

Pioneering transcriptional profiling of C. neoformans during infection of the murine lung (9) and rabbit cerebrospinal fluid (10, 11) by Kronstad, Perfect and coworkers have clearly demonstrated that the expression of many genes related to central carbon metabolism is altered relative to *in vitro* conditions. These labs have gone on to show that specific enzymes involved in glucose metabolism and acetyl-CoA production are critical for either establishment of infection or progression of disease (12, 13). Griffiths et al. demonstrated that ATP-citrate lyase (*ACL1*), an enzyme that converts citrate generated in, and transported from, the mitochondria into cytosolic acetyl-CoA, is required for robust growth in glucose, production of virulence traits, and virulence in the pulmonary mouse model of cryptococcosis (13). In addition, acetyl-CoA synthetase (*ACS1*) was found to be dispensable for growth on glucose but important for replication on nonfermentable carbon sources such as acetate, glycerol, and ethanol (9). Although deletion of *ACS1* reduced virulence in the mammalian model of cryptococcosis (9), it caused delayed mortality relative to the near avirulence of *acl1*Δ mutants (13). Thus, full fitness of C. neoformans during mammalian infection appears to require acetyl-CoA be generated from a variety of metabolic sources.

**FIG 1:**
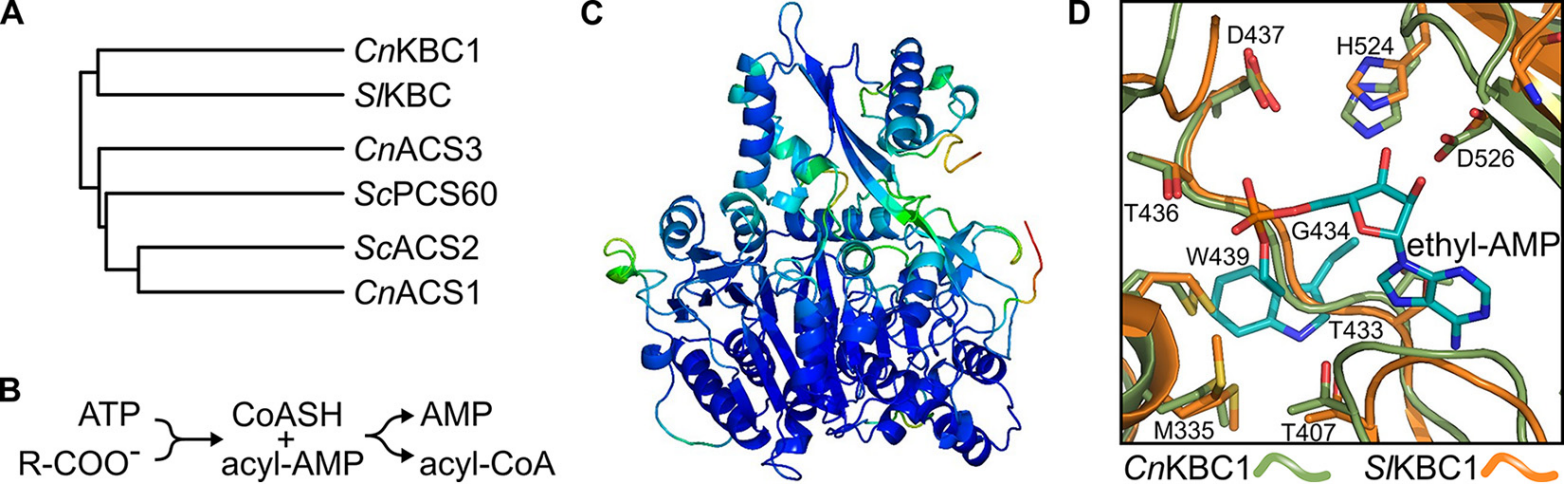
Sequence and structure homology indicate CNAG_02045 encodes a 3-keto-butanoyl-CoA synthetase, *KBC1*. Phylogenetic relationship of ANL-family acyl-CoA synthetases generated using iTOL from a multiple sequence alignment using COBALT (A). Reaction diagram of ANL-family acyl-CoA synthetases (B). AlphaFold2 model of C. neoformans Kbc1 generated using the Colab server and colored by spectrum (red-to-blue with increasing level of confidence) of predicted LDDT (local distance difference test) per residue. Image rendered using PyMol v.2.4.0a0 (C). Overlay of AlphaFold2 model of C. neoformans Kbc1 (green), S. lividans Kbc1 (PDB 4WD1, orange), and key components from *C*. *neoformas* Acs1 (PDB 7KNO, cyan). Ethyl-AMP inhibitor from *Cn*Acs1 shown to demonstrate relative position of the active site. *Cn*Acs1 “Trp wall” residue W439 side chain occupies same position as the backbone atoms for *Cn*Kbc1 (G434) and *Sl*Kbc1 (G422), effectively closing off the pocket to larger substrates (D).

Our interest in acetyl-CoA metabolism in C. neoformans was prompted by the discovery of a small molecule inhibitor of Acs1 that showed antifungal activity and, in particular, synergy with fluconazole against C. neoformans *in vivo* (14). Yeast such as Saccharomyces cerevisiae, Candida albicans and Candida glabrata lack ACL orthologs; instead, they express two copies of ACS, one which is essential (15). As discussed above, C. neoformans lacking *ACS1* are viable. Although the viability of the *acs1*Δ mutant is likely due to the presence of *ACL1*, which is present in other fungi such as Aspergillus spp., or the presence of additional isoforms of *ACS*. Based on the annotation of Hu et al., C. neoformans has two genes related to *ACS1* and, accordingly, these were annotated as *ACS2* and *ACS3* (9). *ACS1* was clearly related to the essential Sc*ACS2* isoform which is localized to the nucleus and cytoplasm (Fig. 1A); *ACS3* was much less related to ACSs and shows sequence similarity to the S. cerevisiae oxalyl CoA synthetase *PSC60* based on BLAST search. CnAcs2 was more closely related to CnAcs1, ScAcs1, and ScAcs2 than Cn*ACS3*. A key distinction between CnAcs1 and CnAcs2 is that CnAcs1 has tryptophan in the conserved substrate binding pocket while CnAcs2 has a glycine (Gly 434). Other members of the **A**cyl-CoA/**N**RPS/**L**uciferase (ANL) family with glycine at this position are acetoacetyl-CoA synthetases that convert the ketone body acetoacetate to acetoacetyl-CoA (16). Acetoacetyl-CoA can then be split to two molecules of acetyl-CoA by acetoacetyl-CoA lyases such as *POT1*/CNAG_00490.

Here, we report that CNAG_02045 is an acetoacetyl-CoA synthetase and propose a revised name of 3-**k**eto-**b**utanoyl-**C**oA synthetase (*KBC1*) that is based on the IUPAC systematic chemical name for its substrate and is consistent with current gene naming conventions. We hypothesized that a tertiary route for acetyl-CoA production through *KBC1* may play a role in virulence and that coordinated regulation of *ACS1*, *ACL1* and *KBC1* is important for the virulence of Cryptococcus neoformans. Consistent with that hypothesis, genetic interaction analysis shows that *KBC1* negatively interacts with *ACS1* to reduce fitness of C. neoformans during infection of the brain. We also find that loss of *ACS1* and *KBC1* increase susceptibility of C. neoformans to fluconazole treatment *in vivo* to a much greater extent than observed *in vitro*. During replication in macrophages, loss of any of the three acetyl-CoA related genes leads to reduced fitness, consistent with the low nutrient status of the phagolysosome. We also show that the expression of *KBC1* and *ACS1* is increased *in vivo* and are coordinately regulated by lipids *in vitro*. These data support the conclusion C. neoformans adapts by utilizing multiple carbon sources to generate the requisite amount of acetyl-CoA for efficient replication.

## RESULTS

### CNAG_02045 is a specific acetoacetyl-CoA synthetase.

As discussed above, the sequence of *ACS2*/CNAG_02045 matches much better with an acetoacetyl-CoA synthetase than an *ACS* (Fig. 1A, Table S1). ANL-family acyl-CoA synthetases (17) catalyze a two-step reaction in which the carboxylic acid is first coupled with ATP to generate an acyl-AMP ester and release pyrophosphate (Fig. 1B). In the second step of the reaction, CoASH condenses with the acyl-AMP to release AMP and the acyl-CoA product. We generated a homology model of CNAG_02045 based on the structure of the bacterial Streptomyces lividans acetoacetyl-CoA synthetase (Fig. 1C, reference 16). Using this model, we compared the predicted substrate binding site of CNAG_02045 to the crystal structure of Acs1 bound to an ethyl-AMP substrate mimic (Fig. 1D, reference 18). In Acs1, W439 acts as a “wall” that limits the size of the acetate binding pocket and prevents larger alkyl substituted carboxylic acids from functioning as substrates (17, 18). CNAG_02045 has a Gly (G433) at the position analogous to W439 and, in contrast, the backbone of the peptide occupies the space where the acetyl group would normally bind. As such the predicted substrate binding site of CNAG_02045 is structurally quite distinct from Acs1. No structures of acetoacetyl-CoA synthetases with substrates or substrate mimics have been reported. Mitchell et al. have proposed that a highly conserved threonine (T436) may function as an H-bond donor to the aceoacetate substrate; however, that hypothesis has not been experimentally tested (16).

10.1128/mbio.01279-22.4 TABLE S1 Alignment matrix of fungal and bacterial acyl-CoA synthetases. The numerical score indicates improved alignment and that Kbc1 is closest to the bacterial acetoacetyl-CoA synthetase *Sl*Kbc. Download Table S1, PDF file, 0.1 MB. Copyright © 2022 Alden et al. 2022 Alden et al. https://creativecommons.org/licenses/by/4.0/ This content is distributed under the terms of the Creative Commons Attribution 4.0 International license.

A 6×-histidine-tagged CNAG_02045 construct was expressed in E. coli and the protein purified by IMAC (Fig. S1A). We used a coupled, continuous assay based on detection of pyrophosphate-release that we previously optimized for CnAcs1 (18) to assay CNAG_02045 for acetoacetyl-CoA synthetase activity (Fig. S1B). We observed robust activity that was dependent on both enzyme concentration (Fig. S1C and D) and the presence of all reaction substrates (Fig. S1E). The K_m_ values for the substrates were: acetoacetate (239 ± 97 μM); ATP (40 ± 4 μM); and CoASH (55 ± 11 μM) (Fig. S1F). We also observed significant substrate inhibition for CoASH (*K_i_* 1130 ± 210 μM).

10.1128/mbio.01279-22.1 FIG S1 (A) Activity detection and assay validation of recombinant *KBC1*. Coomassie gel of verifying size of recombinant *Cn*Kbc1 protein. (B) Reaction diagram of assay using EnzChek pyrophosphate detection kit (Thermo-Fisher). (C) Reaction progression with 2 μg purified Kbc1 shows a long, steady linear range over time. (D) Activity detected by assay increases with increasing *KBC1* concentration. (E) Assay activity is dependent on all substrates of *KBC1* and the enzyme itself being present. (F) Representative Michaelis−Menten curves for all substrates of Kbc1: acetoacetate, ATP, and CoA, respectively. Download FIG S1, PDF file, 0.1 MB. Copyright © 2022 Alden et al. 2022 Alden et al. https://creativecommons.org/licenses/by/4.0/ This content is distributed under the terms of the Creative Commons Attribution 4.0 International license.

**FIG 2:**
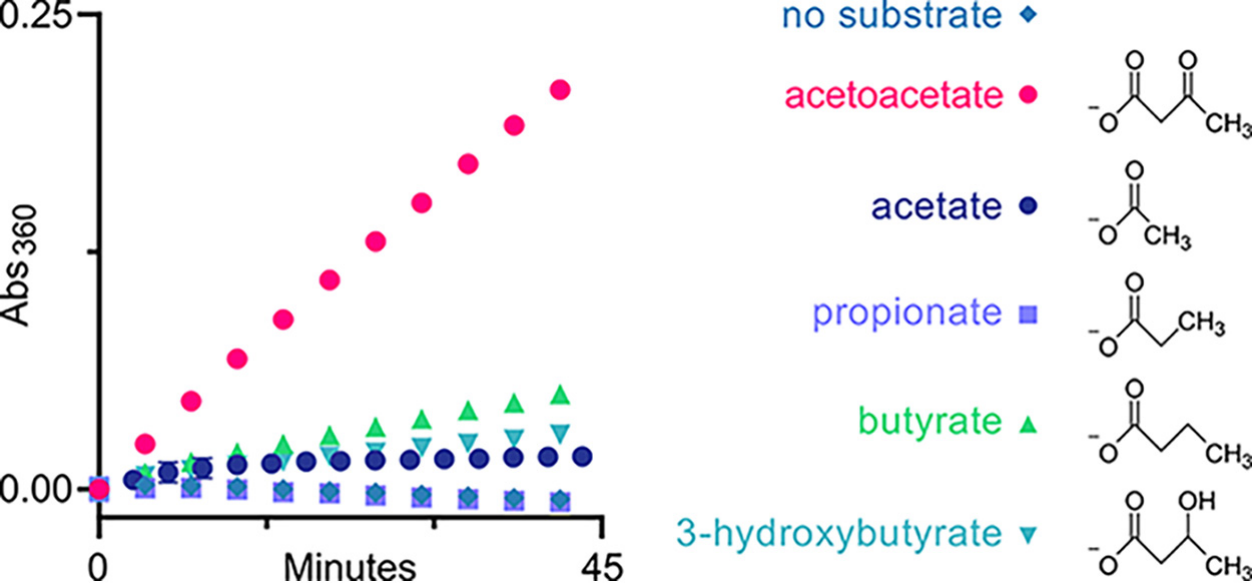
Biochemical characterization validates acetoacetate substrate preference of Kbc1. Kbc1 activity using different substrate substitutions for acetoacetate; all substrates at 10 mM except for acetoacetate at 1 mM.

Despite the presence of the relatively small Gly residue in the putative substrate binding pocket, previously characterized acetoacetyl-CoA synthetases display selectivity for acetoacetate relative to other small-to-medium sized alkyl or aryl carboxylic acids (19). To assess the selectivity of CNAG_02045, we compared the activity of four carboxylic acids at 10 mM to acetoacetate at 1 mM (~5× K_m_). Acetate (C2), propionate (C3), butyrate (C4) and 3-hydroxybutyrate, the reduced form of acetoacetate and also a ketone body, showed minimal conversion under these conditions (Fig. 2). CNAG_02054 is a selective acetoacetyl-CoA synthetase and is unlikely to function as a second acetyl-CoA synthetase. To align the gene name for CNAG_02045 with its biochemical function, we propose changing *ACS2* to *KBC1* which is based on the systematic IUPAC name for its substrate, 3-ketobutanoate, to both keep the three-letter convention and avoid confusion with acetyl-CoA synthetases.

### *ACS1* and *ACL1* appear to be synthetic lethal in C. neoformans.

**FIG 3:**
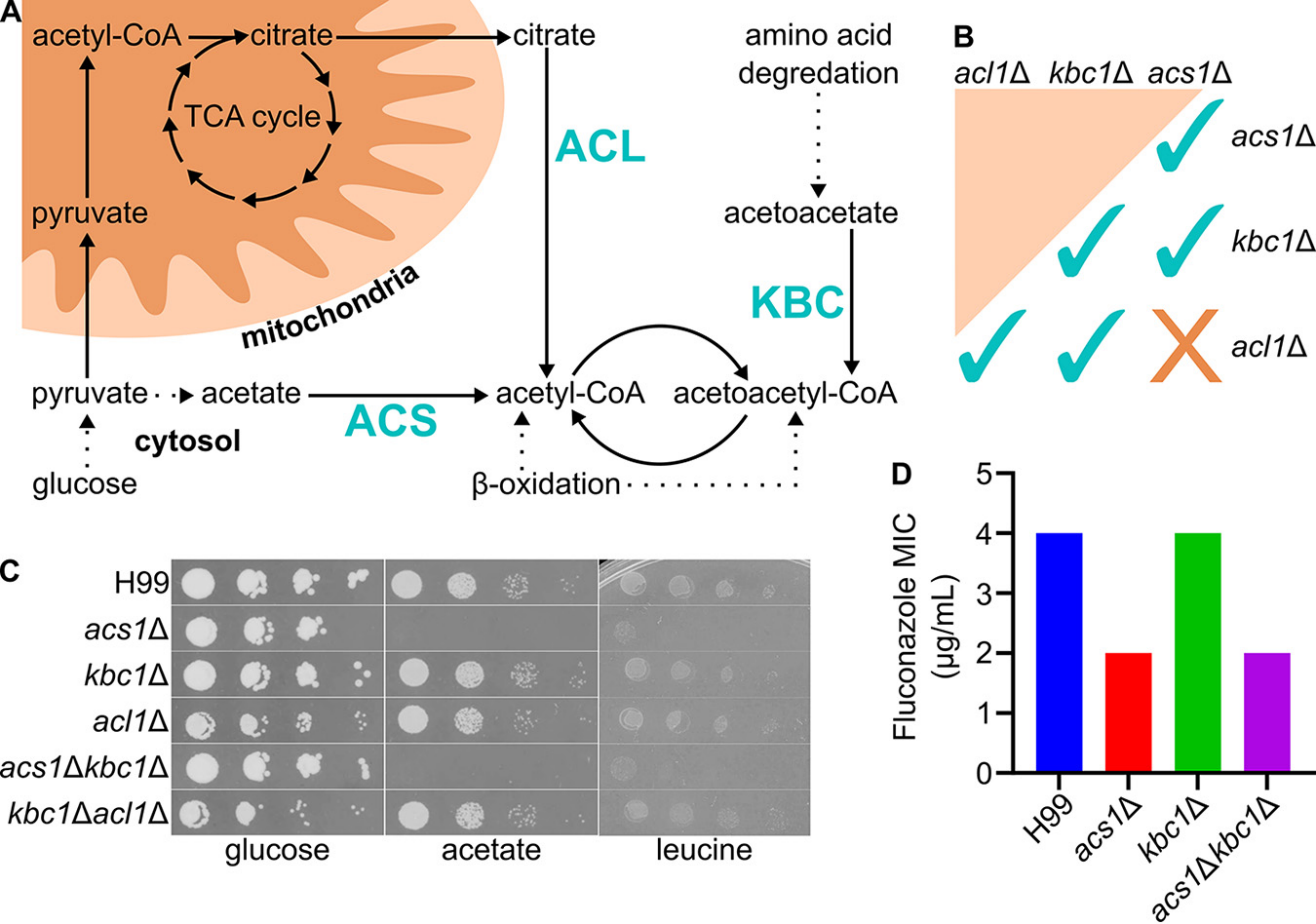
Acetyl-CoA production requires *ACL1* or *ACS1*, but not *KBC1 in vitro*. Diagram of major acetyl-CoA sources in C. neoformans (A). Different single and double deletion mutants made, spots on YPD were imaged after 48 h at 30°C (B). Spot dilutions of all mutants on YNB supplemented with either 2% of glucose, acetate, or leucine as the carbon source, imaged after 72 h at 30°C (C). MICs of fluconazole in RPMI, 72 h at 37°C (D).

Acetyl-CoA is derived from multiple carbon sources (Fig. 3A). Glucose, the preferred carbon source for C. neoformans (13), can be converted to acetyl-CoA by two metabolic pathways (9, 12). In both pathways, glucose is glycolytically converted to pyruvate. In the first pathway, pyruvate is directly converted to acetyl-CoA in the mitochondria by pyruvate dehydrogenase and enters the tricarboxylic acid (TCA) cycle. Acetyl-CoA cannot cross cellular membranes (20) and, thus, the acetyl-CoA equivalent is exported from the mitochondria to the cytosol as citrate. *ACL1* then converts citrate to succinate and one molecule of acetyl-CoA (21). In the second pathway, pyruvate is converted to acetaldehyde by pyruvate decarboxylase. Aldehyde dehydrogenases oxidize acetaldehyde to acetate which is the substrate for *ACS1*.

The oxidation of fatty acids generates one molecule of acetyl-CoA per round of β-oxidation and ultimately generates acetoacetyl-CoA as the final product. Acetoacetate is generated by the degradation of ketogenic amino acids such as leucine. *KBC1* converts acetoacetate to acetoacetyl-CoA and thus these two pathways converge. Acetoacetyl-CoA lyase (*POT1*) converts acetoacetyl-CoA to two molecules of acetyl-CoA. To explore how these pathways function together, we generated a systematic set of double deletion mutants derived from *ACL1*, *ACS1*, and *KBC1* (Fig. 3B). We were able to construct deletions for all combinations except *acl1*Δ *acs1*Δ. Despite multiple attempts, including the generation of a conditional expression allele regulated by the *CTR4* promoter, we were unable to construct a strain lacking both *ACL1* and *ACS1*. This apparent synthetic lethality suggests that these two pathways compensate for one another and are the two dominant acetyl-CoA-generating pathways.

We screened the single and double mutants on a wide range of media and growth conditions. As expected from previously reported studies, strains lacking *ACS1* did not grow when acetate was the sole carbon source (Fig. 3C). Similarly, the *acl1*Δ mutant showed modestly reduced growth on glucose and WT-growth on acetate-containing media. We found no standard growth media or carbon sources in which the *kbc1*Δ mutant showed a growth defect; nor did we observe a synthetic interaction of the *KBC1* deletion with other mutants (Table S1).

We did discover one additional phenotype for the *acs1*Δ deletion strain. As shown in Fig. 3C, both the *acs1*Δ and the *acs1*Δ *kbc1*Δ double mutant has very poor growth when leucine is the sole carbon source. Indeed, WT strains have significantly reduced growth relative to glucose and acetate. Leucine catabolism generates cytosolic acetyl-CoA without the use of mitochondrial enzymes. As such, citrate would be expected to be quite low under these conditions. Both Acl1 and Acs1 are involved in generating nuclear acetyl-CoA for histone acetylation. Acl1 would require the important of citrate for it to carry out this function while Acs1 uses acetate. If citrate is indeed limiting when leucine is the sole carbon source, then Acs1 would be required to carry out the essential process of histone acetylation and its absence would lead to a severe growth defect. We were somewhat surprised that the *acs1*Δ *kbc1*Δ double mutant did not show a synthetic effect under these conditions since leucine leads to one molecule of acetyl-CoA and acetoacetate. However, it appears that the acetoacetate to acetyl-CoA pathway is not essential under these conditions.

One of the critical functions of acetyl-CoA is as a precursor to ergosterol biosynthesis. We, therefore, determined the susceptibility of the double mutants to fluconazole. Interestingly, we did find that the *acs1Δ* mutant was modestly but consistently more susceptible to fluconazole than WT with a 2-fold reduction in MIC under modified CLSI conditions (Fig. 3D). We confirmed this observation using disc diffusion assays (Fig. S2). The deletion of *kbc1*Δ did not affect the MIC. Hu et al. previously reported that the *acs1*Δ mutant showed the same fluconazole susceptibility as WT (9). We suspect that the difference is that Hu et al. determined MICs at 30°C in YNB and YPD medium while we used buffered RPMI and incubated the cells at 37°C. Regardless, the loss of *ACS1* has a modest effect on fluconazole susceptibility *in vitro*.

10.1128/mbio.01279-22.2 FIG S2 Disk diffusion assays of the fluconazole susceptibility of *acs1*Δ, *kbc1*Δ, and *acs1*Δ *kbc1*Δ mutants. Disk diffusion with 20 μg fluconazole on YNB +2% glucose plate, imaged after 72 h at 37°C, diameter of the zone of clearance is in teal, measurement in orange. The images and measurements are representative of three independent replicates. Download FIG S2, PDF file, 0.1 MB. Copyright © 2022 Alden et al. 2022 Alden et al. https://creativecommons.org/licenses/by/4.0/ This content is distributed under the terms of the Creative Commons Attribution 4.0 International license.

### Deletion of *ACS1* and *KBC1* reduces survival in macrophages.

Previously, Hu et al. reported that deletion of *ACS1* had no effect on the three classic C. neoformans virulence traits: growth at 37°C, melanin formation, or capsule formation (9). Similarly, deletion of *KBC1* either alone or in combination with the *acs1*Δ mutation did not affect these phenotypes. We did not examine the deletion of *KBC1* or *ACS1* in combination with *acl1*Δ because the *acl1*Δ mutant shows severe virulence trait phenotypes (12) and therefore it would not be possible to detect additional changes in the double mutants.

**FIG 4:**
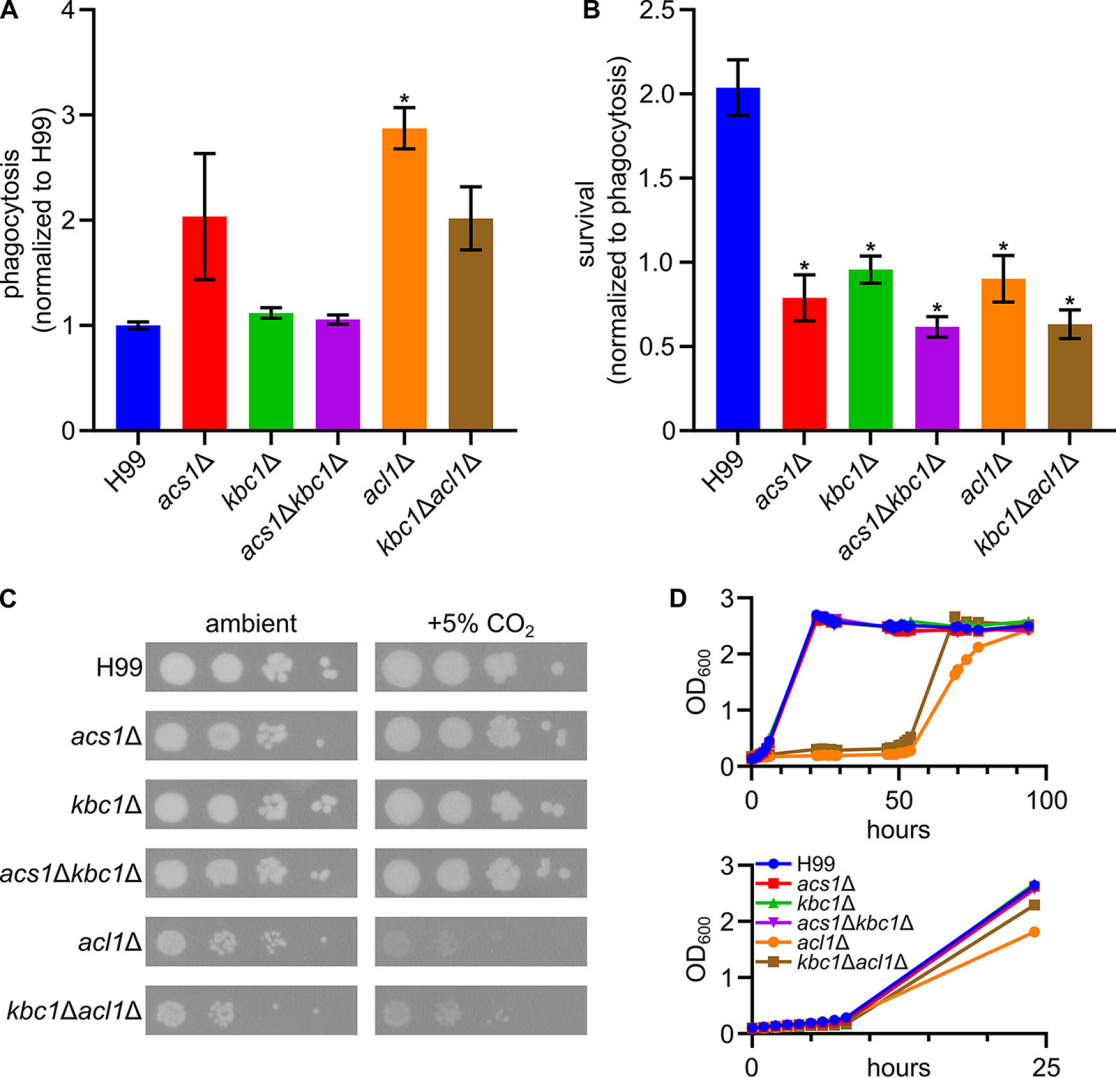
*ACL1*, *ACS1*, and *KBC1* are all required for full fitness in phagocytosed cells. Phagocytosis of different mutants by J774 (MOI 5:1) macrophages normalized to H99 parent strain (A). Survival of phagocytosed mutants in J774 cells 24 h after uptake, normalized to each strain’s phagocytosis levels (B). Spot dilutions of *acl*Δ mutants show a growth defect on RPMI-MOPS with 5% CO_2_, plates imaged after 96 h at 37°C ± 5% CO_2_ (C). Growth curves show *acl*Δ mutants have 48 h lag phase in RPMI-MOPS after YPD overnights; however, when hour 69 samples were taken and back diluted, there were no growth differences between any strain (D). Analysis done using a one-way ANOVA; *, *P* < 0.05 compared to H99.

A critical aspect of C. neoformans pathogenesis is its ability to replicate within the macrophage (3). The macrophage is thought to be a nutrient-poor niche (22) and, therefore, we hypothesized that it may place demands on central carbon metabolism that are not well-replicated *in vitro* (23). We, therefore, tested the ability of the double mutants to replicate within the murine macrophage-like cell line J774. Except for the *acl1*Δ mutant, all strains were phagocytosed by J774 cells similarly to the H99 reference strain; *acs1*Δ and the *kbc1*Δ *acl1*Δ showed a trend toward increased phagocytosis but the differences were not statistically significant (Fig. 4A). Griffiths et al. had found that there was no difference in uptake of their *acl1*Δ mutants. It is likely that the discrepancy between these results is due to differences in the assays used to characterize phagocytosis. Specifically, Griffiths et al. used a microscopy-based assay of phorbol-myristate acetate-stimulated J774 cells at a multiplicity-of-infection (MOI, yeast to J774) of 1:1 (12). Our data are based on the fungal burden determined by quantitative plating of J774 cell lysates after incubating at an MOI of 5:1 without prestimulation. We speculate that the prestimulation and the low MOI is likely to reduce the ability of that assay to identify subtle changes in phagocytosis.

Although Griffiths et al. did not observe increased phagocytosis (12), the *acl1*Δ mutant is deficient in capsule formation and capsule is generally considered to be anti-phagocytic (24). However, both of our assays used anti-capsular antibodies in the opsonization step. We also considered alternative explanations related to acetyl-CoA metabolism and this led to the hypothesis that decreased acetyl-CoA generation in the *acl1*Δ might affect chitin biosynthesis. Glucosamine-6-phosphate is acetylated early in chitin biosynthesis in an acetyl-CoA requiring step and overall chitin may be reduced in the absence of *ACL1* or other acetyl-CoA generating enzymes. In Candida albicans, reduced chitin has been associated with increased phagocytosis (25). To test this hypothesis, we stained H99 with calcofluor white and quantified the differences in staining between the mutants using Image J. Although the *acs1*Δ *kbc1*Δ mutant had slightly elevated levels of staining, there was no correlation between chitin and phagocytosis (Fig. S3). As such, the most likely explanation for the increased phagocytosis of *acl1*Δ mutants observed in our experiments is an effect of reduced capsule formation.

10.1128/mbio.01279-22.3 FIG S3 Chitin levels do not correlate with differences in phagocytosis. Strains were grown overnight, stained with 10 μg/mL of calcofluor white, and imaged. Images were analyzed in ImageJ to look at intensity of staining. Analysis done using a one-way ANOVA with Tukey post hoc and adjustment for multiple comparisons. H99 showed a statistically significant difference from *acs1*Δ *kbc1*Δ (*P* < 0.001, Cohen‘s D = 0.78) but all other strains were not different than the reference. Download FIG S3, PDF file, 0.1 MB. Copyright © 2022 Alden et al. 2022 Alden et al. https://creativecommons.org/licenses/by/4.0/ This content is distributed under the terms of the Creative Commons Attribution 4.0 International license.

Next, we determined the fungal burden of J774 cells 24 h after phagocytosis of H99 a*cs1*Δ, *kbc1*Δ, *acl1*Δ, *acs1*Δ *kbc1*Δ, and *acl1*Δ *kbc1*Δ mutants (Fig. 4B). The fungal burden was normalized to the fungal burden of J774 cells immediately after phagocytosis. As expected, H99 cells underwent replication within the J774 cells (relative survival 2, Fig. 4B). In contrast, none of the mutants were able to replicate during this time course (relative survival ~0.6-1). Although the two double mutants had the lowest fungal burden (~60%), there were no significant differences between these mutants and their corresponding single mutants. These data indicate that, although the mutants failed to replicate within the phagolysosome, the majority of cells remained viable. This observation is consistent with a model in which the low nutrient status of the phagolysosome requires the functioning of all acetyl-CoA-generating pathways in order for the cells to efficiently replicate. Although there is a slight reduction in the fungal burden for some of these mutants, it is not sufficiently significant to support the alternative model that the mutants are more susceptible to the antifungal properties of the phagolysosome. Furthermore, our data suggests that the stringency of the nutrient stress within the phagolysosome is such that loss of any of the three acetyl-CoA generating pathways leads to reduced fitness in this key C. neoformans niche.

Our initial characterization of the effect of the acetyl-CoA related mutants on growth under different nutritional conditions focused on specific carbon sources but did not include media that mimics host-like conditions. Tissue culture media, such as the RPMI used in the macrophage experiments, is low nutrient compared to standard media used to grow C. neoformans in the lab. As shown in Fig. 4C, the *kbc1*Δ *acl1*Δ mutant shows a modes growth phenotype on solid RPMI in ambient carbon dioxide at 37°C. The *acl1*Δ mutant had a modest growth defect that was increased by the deletion of *KBC1* but not *ACS1* under ambient concentrations of carbon dioxide. The growth defects of the *acl1*Δ and *acl1*Δ *kbc1*Δ mutants were exacerbated significantly at host concentrations of carbon dioxide (5%), indicating that *ACL1* is critical for the cells to tolerate the stress of elevated carbon dioxide.

However, the results of these assays do not provide an explanation for the fact that loss of any one of the three acetyl-CoA generating enzymes prevents replication within J774 cells. We, therefore, examined the growth curves of the mutants to determine if the mutants cause more subtle growth effects that were not apparent using the spot dilution assays. Consistent with Griffiths et al., we found that stains lacking *ACL1* displayed a prolonged lag phase (12) and then underwent log phase growth with a slope similar to WT (Fig. 4D). When *acl1*Δ cells were harvested, diluted, and then returned to incubation, they showed growth characteristics similar to the H99 and the other mutants (Fig. 4D, lower panel). It is possible that this lag phase contributes to the inability of *ACL1* mutants to replicate within the macrophage but does not explain the phenotypes of the other mutants.

### *KBC1* and *ACS1* expression is increased during infection and host-like *in vitro* conditions.

**FIG 5:**
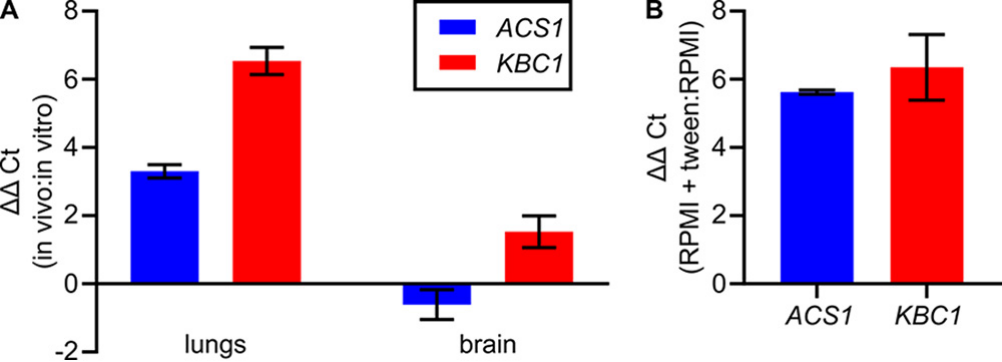
*ACS1* and *KBC1* expression is increased *in vivo* and *in vitro* in the presence of fatty acid. *In vivo ACS1* and *KBC1* expression from lungs recovered 17 days after intranasal inoculation and brains recovered 8 days after intravenous inoculation compared to *in vitro* cultures grown to early log phase in RPMI at 30°C (A). *ACS1* and *KBC1* expression is induced by the addition of a soluble lipid (Tween 20) to RPMI (B).

To further characterize the interplay between the different acetyl-CoA-generating enzymes, we examined their relative expression levels. As discussed above, *ACS1* has previously been shown to be expressed at much higher levels in the mouse lung than in standard laboratory culture conditions, while *ACL1* expression is relatively stable (10). More recently, *KBC1* was also found to be highly expressed in CSF both *in vivo* and *ex vivo* (11). These experiments used large-scale methods and, therefore, we were interested in confirming them and looking directly in infected brain tissue using RT-PCR. We also examined tissue at later time points than Hu et al. had used (9). Consistent with previous results, *ACS1* expression is much higher in infected lung (inoculated intratracheally) than in RPMI (Fig. 5A). Similarly, *KBC1* expression is also upregulated dramatically in the lung. *ACS1* is not significantly upregulated at the time point we examined in the brain after intravenous infection. However, *KBC1* expression in the brain is much higher than in RPMI culture. These data suggest that central carbon metabolism is significantly different during infection compared to *in vitro* conditions.

Based on these findings, we examined a number of host-like *in vitro* conditions (e.g., RPMI, RPMI + 5% CO_2_) to identify those that induce expression of *KBC1* and *ACS1* but were not initially successful at doing so. However, we reasoned that both the lung and brain have relatively high amounts of lipid. To test if lipids induce expression of *KBC1* and *ACS1*, we supplemented RPMI with Tween 20 as a soluble source of fatty acid. As shown in Fig. 5B, this increased expression of both *KBC1* and *ACS1* relative to RPMI alone albeit not to the extent that we observed *in vivo*. This observation suggests that, in the presence of fatty acids, multiple pathways for acetyl-CoA generation are induced and that may contribute to the increased expression of *KBC1* and *ACS1 in vivo*. We did not, however, observe a significant growth defect in the *kbc1*Δ, *acs1*Δ, or *kbc1*Δ *acs1*Δ double mutant in RPMI supplemented with Tween 20 relative to RPMI alone; however, the lipids are not the only carbon source under these host-like conditions.

### Loss of both *ACS1* and *KBC1* reduces fitness during CNS infection and increases susceptibility to fluconazole.

**FIG 6:**
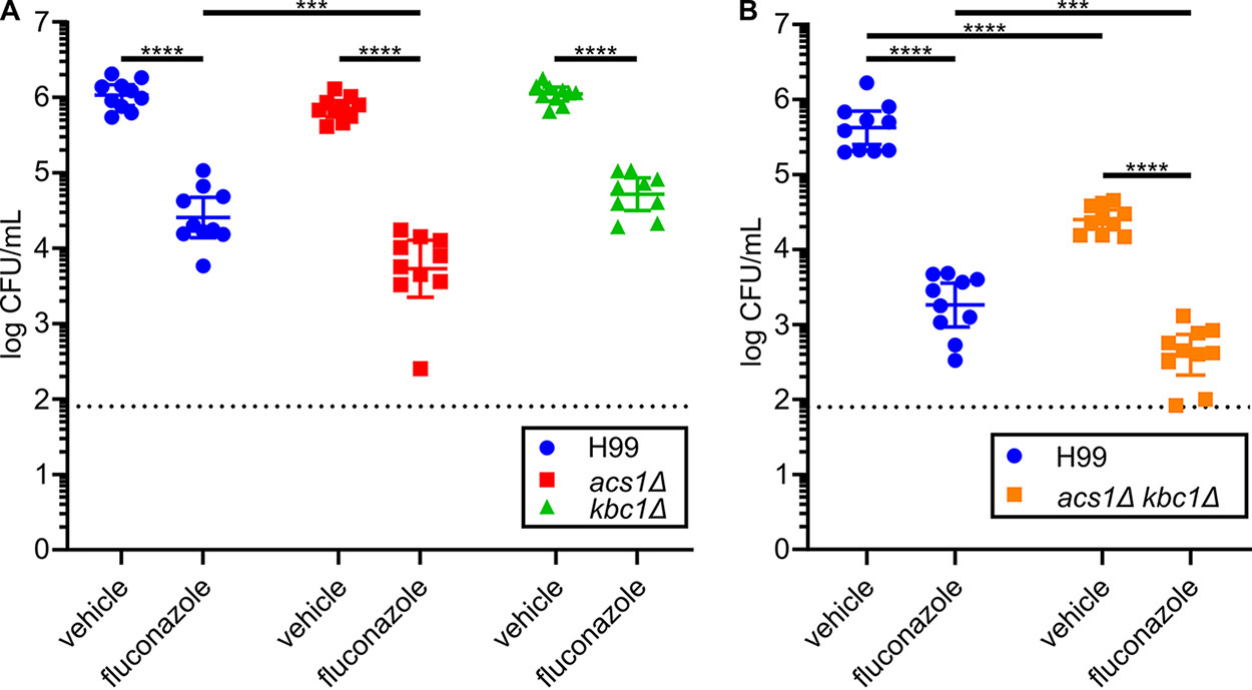
Alternative Pathways for acetyl-CoA generation are required for full fitness during brain infection and affect *in vivo* fluconazole susceptibility. CD-1 mice were inoculated with either the *acs1*Δ or *kbc1*Δ mutant and treated with either 125 mg/kg fluconazole or vehicle for 2 days when their brains were harvested, homogenized, plated, and growth at 30°C for 48 h before counting (A). The experiment was repeated with the *acs1kbc1* double mutant (B). Analysis done using a two-way ANOVA; ***, 0.0001 < *P* < 0.001; ****, *P* < 0.0001.

The increased expression of *ACS1* and *KBC1* during infection suggested that they may play a more important role in the fitness of C. neoformans *in vivo* compared to *in vitro*. Furthermore, these mutants showed defects in fitness within macrophages. Since the *acs1*Δ mutant is modestly more susceptible to fluconazole, we were also interested to see if this phenotype was manifest during infection. Hu et al. had already shown that deletion of *ACS1* led to reduce virulence in a pulmonary infection model of cryptococcosis (9); deletion of *KBC1* in the *acs1*Δ background did not show any additional effects (data not shown). We, therefore, asked if the genes might interact during disseminated infection to the CNS. As shown in Fig. 6A, the fungal brain burden of mice infected with neither the *acs1*Δ nor *kbc1*Δ mutant differed from those infected with wild type. In the presence of fluconazole, however, the *acs1*Δ mutant was more susceptible to fluconazole *in vivo* than either WT or the *kbc1*Δ mutant by approximately 0.5 log_10_ CFU/mL.

In contrast to the two single mutants (Fig. 6B), the fungal burden of the *acs1*Δ *kbc1*Δ double mutant was reduced by >2 log_10_ CFU/mL, indicating that these two acetyl-CoA-generating pathways likely compensate for one another during infection of the CNS. In the presence of fluconazole, the fungal burden of the double mutant is further reduced to near the limit of detection of the infection assay. Taken together, these data clearly indicate that acetyl-CoA biosynthesis is dependent upon multiple pathways during CNS infection in a manner that could not be predicted from *in vitro* characterization. Furthermore, these data also indicate that the metabolic pathways required for pulmonary infection are not necessarily the same as those required for disseminated infection of the CNS.

## DISCUSSION

Acetyl-CoA is one of the most fundamental molecules in biology, in general, and in carbon metabolism, in particular. Fungi can be distinguished into two groups based on the types of enzymes that generate acetyl-CoA (9, 12, 15). The first group relies on ACS localized to nucleus/cytoplasm and mitochondria to generate non-pyruvate decarboxylase-derive molecules acetyl-CoA. This group includes Saccharomyces cerevisiae and Candida albicans. In these yeast species, acetyl-CoA synthetases are essential (26). The second group of fungi generate acetyl-CoA through two enzymes: ACS and ACL. C. neoformans and Aspergillus spp. are two examples of fungi with both ACS and ACL (27). In humans, ACL is the major source of acetyl-CoA while ACS contributes significant acetyl-CoA only under severe stress or in the case of tumor cells where it can become the major acetyl-CoA generating enzyme (28). ACL is essential in mammals (29).

Our group has identified a molecule, AR-12, that inhibits fungal ACS and has potent, broad spectrum antifungal activity, including fungi that have both ACS and ACL (14). In mouse model efficacy studies, AR-12 improves the activity of fluconazole against C. neoformans. Since ACS is typically not essential in fungi with both ACS and ACL enzymes, we took a genetic interaction approach to investigate the mechanistic basis for the antifungal activity of AR-12. We have been unable to generate double mutants of *ACS1* and *ACL1* or conditional expression strains of either gene in the absence of the other. Although not definitive, these observations strongly suggest that these two genes are synthetically lethal. As reported previously by Kronstad lab (12), mutants lacking *ACL1* have increased expression of *ACS1*, suggesting that these two enzymes may compensate for one another and further supporting the assertion that loss of both enzymes would be lethal.

Acetyl-CoA equivalents generated in the mitochondria by the TCA cycle are exported from to the cytosol as citrate which is then converted to acetyl-CoA and oxaloacetate by Acl1. As such, conditions that require Acl1-generated acetyl-CoA are also dependent upon mitochondrial function. In our characterization of the mode of action for AR-12, we discovered that AR-12 rapidly depolarizes the mitochondrial membrane (14), leading to a loss of the proton-motive force. The electron-transport chain is required to replenish NAD^+^/FAD^+^ for proper TCA function and, thereby, deliver citrate to the cytosol for conversion to acetyl-CoA by Acl1. Consequently, AR-12 may induce the equivalent of a reduction in Acl1 function by interfering with the ability of the cell to generate citrate through the TCA cycle. Therefore, we propose that the broad-spectrum activity of AR-12 may be due to the direct inhibition of Acs1 coupled with the indirect reduction in substrate for Acl1, leading to the equivalent of a synthetic lethal interaction.

Cytosolic acetyl-CoA is required for lipid biosynthesis, including ergosterol. Consistent with this requirement, Griffiths et al. found that *acl1*Δ deletion mutants were hypersusceptible to fluconazole (12). Under the conditions that they used (YPD and YNB at 30°C), *acs1*Δ mutants did not show significant changes in susceptibility; at 37°C on buffered RPMI medium, *acs1*Δ mutants showed modestly increased susceptibility to fluconazole. Deletion of *KBC1* in the *acs1*Δ background did not further sensitize the strain to fluconazole. One of the striking findings of our work is that the effect of these mutations on fluconazole susceptibility is much more pronounced *in vivo* than it is *in vitro* (Fig. 6). The *acs1*Δ mutant has only a 2-fold change in MIC *in vitro* but the brain burden of *acs1*Δ-infected mice is reduced by 5-fold. Again, in contrast to *in vitro* conditions, deletion of *KBC1* in the *acs1*Δ background further increased the fluconazole susceptibility to the point where the infection was nearly cleared. These data indicate that during brain infection ergosterol synthesis is likely to be dependent on all three acetyl-CoA-generating enzymes.

Further supporting this conclusion, the *kbc1*Δ *acs1*Δ double mutant has a fitness defect in the absence of antifungal drugs. Deletion of *ACL1* is already known to severely reduce fitness *in vivo*, indicating it plays a key role in the generation of acetyl-CoA *in vivo*. However, the fitness defect of the *kbc1*Δ *acs1*Δ strain indicates that Acl1 is not sufficient to be the sole source of acetyl-CoA during brain infection. Although the mechanistic basis for this observation remains unclear, our expression studies indicate that the expression of *KBC1* and *ACS1* is increased during infection and under host-like *in vitro* conditions supplemented with lipid. Taken together, these data suggest that C. neoformans may be generating acetyl-CoA from multiple pools of carbon during infection.

The macrophage is another key niche for C. neoformans and the ability to replicate within the phagolysosome is a critical part of the mechanism of pathogenesis (3, 22). The phagolysosome is widely regarded to be a nutrient-poor environment and phagocytosed yeast show transcriptional profiles that support this model (23). Again, Griffiths et al. found that genes required for acetyl-CoA synthesis were strongly induced (12), including *ACL1*. Consistent with these findings, they also found that the *acl1*Δ mutant showed reduced replication within macrophages. Although neither *ACS1* nor *KBC1* was induced in macrophages, both genes are also required for C. neoformans to replicate in macrophages. It is important to emphasize that loss of these enzymes did not lead to significant loss of viability within the macrophages, further supporting a model whereby reduced nutrient availability prevents replication. Accordingly, the macrophage environment appears to be the most stringent in terms of the number of pathways required to maintain sufficient acetyl-CoA production. The utilization of multiple carbon metabolism pathways during macrophage infection by C. neoformans is consistent with recent observations reported for C. albicans as well (22, 23).

The only *in vitro* genetic interaction that we observed was between *ACL1* and *KBC1* on RPMI medium at ambient carbon dioxide. The main carbon sources present in this medium are glucose (0.5%) and amino acids. *ACL1* deletion reduces growth in the presence of this low concentration of glucose on multiple media and we suspect that the further reduction in growth may be because Kbc1 is part of the pathway converting amino acids (e.g., leucine) to acetyl-CoA. It is also interesting that host levels of carbon dioxide further exacerbate the fitness defect of *acl1*Δ on host-like media. Our group has recently shown that host concentrations of carbon dioxide is an independent stress with relevance to virulence (8). Specifically, environmental strains with low mammalian virulence do not tolerate host concentrations of carbon dioxide while clinical strains do. The decreased fitness of the *acl1*Δ mutant indicates that carbon dioxide increases the cell’s dependence on acetyl-CoA derived from the mitochondria. Our observation of this phenotype provides additional insight into the basis for the profound virulence defect observed for strains lacking *ACL1*.

Price et al. previously showed that the glycolytic pathway was critical for disease but not persistence within the CNS (13). In the absence of glycolysis, alternative pathways to the generation of acetyl-CoA would be required. Our data strongly suggest that a combination of Acs1 and Kbc1 provide this carbon flow and, thereby, support the replication of C. neoformans within the CNS. As such, our observations for the role of Acs1 and Kbc1 fit well with existing data and provide additional insights into the metabolic requirements of C. neoformans during infection.

In summary, our genetic interaction approach to the study of three enzymes involved in acetyl-CoA production indicate that C. neoformans uses a variety of carbon sources and pathways to generate this critical metabolite during infection. Although C. neoformans is well-known to have a strong preference for glucose utilization and is an obligate aerobe, it appears to also have significant metabolic versatility which, in turn, is particularly important during infection of macrophages and the central nervous system.

## MATERIALS AND METHODS

### Strains, media, and growth.

Strains from Cryptococcus neoformans H99 background were maintained in glycerol stocks kept at −80°C and recovered on YPD plates (1% yeast extract, 2% peptone, 2% dextrose, 2% agar). Experiments performed with the strains (with the exception of *in vivo* experiments, which were grown for ~48 h) used cultures grown overnight shaking at 220 rpm at 30°C in 2 mL liquid YPD (1% yeast extract, 2% peptone, 2% dextrose). For growth on plates, YNB plates (0.17% yeast nitrogen base without amino acids, 0.5% ammonium sulfate, 2% agar) were supplemented with 2% m/v of a carbon source: glycerol, acetate, glucose, or BHB; overnights of desired strains were then diluted in liquid YPD to an OD_600_ of 1.0 and serial diluted 10-fold down to 0.001 OD_600_, all four dilutions of each strain were plated using a replica spotter onto the medium of choice. Incubations under a carbon dioxide atmosphere (5%) were carried out in a standard tissue culture incubator at 37°C.

### Strain construction.

*ACS1* deletions were performed by replacing the gene locus with the nourseothricin resistance marker using biolistics as previously described (30). To generate *KBC1* deletions in H99 and Δ*acs1*, both strains were transformed using the protocol and CRISPR-TRACE system previously described, with minor modification (31). The neomycin selective resistance marker was used to replace the *KBC1* locus using electroporation with a Bio-Rad MicroPulser on the “Pic” setting. The deletion construct was designed to leave the 3′ noncoding region of CNAG_02406 intact. Strains were passaged three times on nonselective media before replating on selective media to check for stable marker integration. Selected mutants from both backgrounds were confirmed by southern blot. *ACL1* deletions were generated in the H99 and Δ*kbc1* strains using the Cryptococcus optimized Cas9 system with 50 bp homology as previously described using a hygromycin resistance marker (32). The protocol for transformation was the same as before and selected mutants were again confirmed via southern blot.

### Enzyme expression and purification.

Cryptococcus neoformans *KBC1* was cloned into the NdeI/XhoI site of the pET15-b expression vector and transformed into Escherichia coli strain BL21 with ampicillin selection. An overnight starter culture was prepared in LB broth with antibiotic and grown at 37°C at 220 rpm overnight. The next day, the overnight was diluted 1:100 in LB medium with antibiotic and grown to OD_600_ of 0.5 to 0.8, then induced with 1 mM isopropyl-β-D thiogalactopyranoside (IPTG) for 2 h. Pelleted cells were resuspended in lysis buffer (1 mM PMSF, 1 mM DTT, 0.25 μL/L benzonase, 1 mg/mL lysozyme, 10 mM tris-HCl pH 7.5, 20 mM imidazole, 1 mM MgCl_2_, 200 mM NaCl) on ice and sonicated for two cycles of 3 min on, 3 min rest. Pelleted lysate was then mixed with washed Ni beads and transferred to a column. Wash buffer (20 mM imidazole, 20 mM tris-HCl pH 7.5, 150 mM NaCl) was run over the column, followed by elution buffer (300 mM imidazole, 20 mM tris-HCl pH 7.5, 150 mM NaCl). The elution was collected and placed in dialysis cartridges in Kbc1 enzyme buffer (200 mM NaCl, 50 mM tris-HCl pH 7.5, 1 mM MgCl_2_, 10% glycerol, 1 mM DTT) stirring overnight at 4°C. Purified Kbc1 was stored at −80°C.

### Acetoacetyl-CoA synthetase assay.

Kbc1 activity was detected using our assay previously described for Acs1 activity except sodium acetoacetate was used in place of sodium acetate (18). Michaelis-Menten constants were determined for the acetoacetate, CoA, and ATP substrates by first determining the optimal concentrations of these substrates so that they would be in excess without, in the case of CoA, being high enough to inhibit the reaction. Substrates provided in excess allow the apparent Km to closely approximate the actual Km. The EnzChek Pyrophosphate assay kit (Thermo) was used with reagents prepared by manufacturer’s standards, and with 4 mM MgCl_2_, 10 mM DTT, 4.5 μg/mL Kbc1, 100 μM CoA, and 200 μM ATP per 50 μL reaction. The reagents were mixed and aliquoted at room temperature, allowed to incubate for 15 min at 37°C to mop background phosphate contamination, then acetoacetate (final concentration of 1 mM) was added to the reaction, and the plate was read continuously for 40 min at 37°C in a SpectraMax i3X Multi-Mode plate reader (Molecular Devices) at absorbance 360 nm. To test possible substrates, a dilution series of propionate, butyrate, 3-hydroxybutyrate, or acetate was added in place of acetoacetate.

### Assays of capsule formation and melanin production.

H99, Δ*acs1*, Δ*kbc1*, and Δ*acs1*Δ*kbc1* strains were grown overnight in YPD. The next day, 1 mL of each culture was spun down, washed twice in PBS, resuspended in 1 mL of RPMI/MOPS (pH 7.4), counted using a digital hemocytometer (Countess), and diluted to 7.5 × 10^5^ cells per mL RPMI/MOPS. Diluted cells were then aliquoted 100 μL per well of a 96-well plate and sealed with a permeable membrane and incubated at 37°C in 5% CO_2_. Time points were taken at 24, 48, and 72 h, one well of each strain was recovered, spun down, supernatant removed, resuspended in 20 μL PBS, mixed 1:1 with India ink and imaged at 40× for capsule formation. For melanin production, overnights were diluted 1:100 in dH_2_O, counted using a Countess II FL (Invitrogen), and diluted to 1 × 10^7^ cells per mL in water. 10-fold serial dilutions in water to 1 × 10^3^ cells per mL were spotted onto l-DOPA plates using a replica spotter. Plates were incubated at 30°C for 2 days and then imaged.

### Antifungal drug susceptibility assays.

MIC assays were performed using a slightly modified CLSI standard method. Fluconazole was diluted 2-fold in DMSO and added to RPMI/MOPS so that the final DMSO concentration in each well was 1.28%. Overnights of selected strains were washed 3 times in PBS, resuspended in RPMI/MOPS, counted using a Countess II FL (Invitrogen), and diluted to 1 × 10^5^ cells per mL in RPMI/MOPS. Diluted cells were added to triplicate wells for each drug dilution such that each well had ~1000 cells and incubated at 37°C for 72 h before visual inspection. Following CLSI guidelines, the MIC was defined as the lowest concentration of drug that resulted in a clear well. Disk diffusion assays were done by taking 1 mL of overnights, washing 3 times in PBS, resuspending in 1 mL PBS, then diluting 1:100 in PBS. The 1:100 dilution was spread on top of a YPD plate using a sterile swab to achieve a lawn of cells on the entirety of the plate. Sterile filter paper dots were then added on top of the lawns and 20 μL of desired drug or control was carefully pipetted on the dot and allowed to sink in. Plates were then incubated at 37°C for 72 h and imaged. All assays were performed in triplicate.

### Gene expression analysis by quantitative reverse transcription-PCR.

For *in vitro* expression, 1 mL samples were taken in log phase, OD_600_ = ~0.3 to 0.65, from liquid cultures (with the exception of RPMI/MOPS + Tween 20 for H99, H99Δ*acs*, H99Δ*kbc*, and H99Δ*acs*Δ*kbc*, which were taken at stationary phase). With the MasterPure Yeast RNA purification kit (catalog number MPY03100), RNA was extracted using the manufacturer’s protocol. Pelleted cells were vortexed, mixed with 300 μL extraction reagent with 1 μL proteinase K, incubated at 70°C for 12 min with vortexing every 4 min, then placed on ice for 3 to 4 min. Samples were mixed with 175 μL MPC protein precipitation reagent and then were centrifuged for 10 min at 4°C and 12,000 rpm. Supernatants were transferred and mixed with 500 μL isopropanol, spun down again, rinsed with 70% ethanol twice and RNA was suspended in 35 μL TE buffer. Concentration was measured at 260 nm, and RNA was then converted to cDNA using iScript cDNA Synthesis kit (Bio-Rad catalog number 170-8891), with 4 μL 5× iScript reaction mix, 1 μL reverse transcriptase, 14 μL water, and 1 to 10 μL of RNA (so that there was ~0.4-1 μg RNA) per reaction.

For *in vivo* expression, A/J mice, 3 per condition, were inoculated with H99 1 × 10^4^ intravenously with brains harvested after 8 days or inoculated 5 × 10^4^ intranasally with lungs harvested after 17 days. Organs were lyophilized overnight and then bead beaten with zircon/silica beads for 45 s. 1 mL triazole reagent was added and tubes were incubated at room temperature for 10 min then spun at 10k rpm for 5 min at 4°C. The clarified layer was transferred to a new tube where 200 μL chloroform was added and samples were incubated for 5 min at room temperature then spun at 12k rpm for 15 min at 4°C. The top layer was transferred to a gDNA removal column from Qiagen RNeasy plus kit (Qiagen, catalog number 74134) and the manufacturer’s instructions were then followed from that point. Samples were eluted in 50 μL water, diluted 1:10, and quantified. ~500 ng RNA was used to make cDNA with the same iScript cDNA synthesis kit; the cDNA was then diluted 1:1 in water.

Quantitative reverse transcription-PCR was done with cDNA using TEF1 as a housekeeping gene, *ACS1*, *KBC1*, and *ACL1* using IQ Sybr green supermix (Bio-Rad catalog number 170-8882). Each sample & condition was in technical triplicate and biological duplicate, with 10 μL of the Syber green supermix, 0.25 μM each respective primer, 7.5 ng/μL of cDNA from the *in vitro* samples or 2 μL of the 1:1 dilution from the *in vivo* cDNA, and 8 (*in vitro*) or 7 (*in vivo*) μL of water was in each reaction well.

### C. neoformans–macrophage interactions.

J774 cells were seeded onto a 96-well plate at 2.25 × 10^5^ cells per well and incubated at 37°C with 5% CO_2_ for 24 to 48 h until 80 to 90% confluent. Once they are confluent, standard overnight cultures of the desired yeast strains were washed twice with PBS, counted with the Countess II FL (Invitrogen), and diluted to 1.12 × 10^7^ CFU/mL in PBS for an MOI of 5:1. Each strain is then opsonized with antibody 18b7 (2 μg/mL final) and incubated at 37°C for 1 h with rotation. After opsonization, 80 μL of cells per well were added to the two prepared J744 plates, each with 6 technical replicates. Plates were incubated for 80 min and then washed to remove cells that had not been endocytosed. To determine phagocytosis from the first plate, macrophage cells were lysed with 0.1% cold triton X for 10 min while rocking before plating. To then determine how well those that had been phagocytosed survived, J774 growth medium was added to the second washed plate and left for 24 h at 37°C with 5% CO_2_ and then lysed with 0.1% cold triton X for 10 min while rocking before plating. For plating, cells were serially diluted 10-fold down to 1:1000. All four dilutions (full down to 1:1000) for each well were plated on YPD with 3 × 10 μL spots per dilution and grown for 72 h at 30°C before counting CFU.

### Estimation of cell wall chitin content.

Strains were grown overnight in YPD, washed twice and resuspended in PBS, then stained with Calcofluor white for a final concentration of 10 μg/mL, and incubated for 20 min in the dark. Cells were then washed twice again with PBS and imaged. Five images with at least 5 cells each were taken of each strain. Images were then analyzed in ImageJ.

### Mouse infection model of cryptococcosis.

All animal experiments were carried out in compliance with approval from the University of Iowa Institutional Animal Care and Use Committee (IACUC). For fluconazole susceptibility, 50 mL cultures of strains were grown for 48 h (YPD, 30°C, shaking) and then used to inoculate female CD-1 mice via tail vein injection with 200 μL containing 3.8 × 10^5^ cells. 20 mice were inoculated for with each strain, 10 to be treated with 125 mg/kg fluconazole, 10 to receive the vehicle. Mice were monitored and treatments of fluconazole or vehicle were given via intraperitoneal injection every 24 h starting about 1 h after the initial inoculation. After 3 treatments, the mice were euthanized with CO_2_ and their brains homogenized in 1 mL PBS and serially diluted 10-fold down to 1:10,000. Dilutions of 1:10 down to 1:10,000 for each organ were plated on YPD with 6 × 10 μL spots per dilution and plates were grown at 30°C for 48 h and then CFU were counted.

10.1128/mbio.01279-22.5 TABLE S2 Growth of acetyl-CoA related deletion mutants on various carbon sources. Plates were incubated at 30°C for24 to 96 h. ND, not determined; ++, regular growth; +, reduced growth; -, no growth. Download Table S2, PDF file, 0.1 MB. Copyright © 2022 Alden et al. 2022 Alden et al. https://creativecommons.org/licenses/by/4.0/ This content is distributed under the terms of the Creative Commons Attribution 4.0 International license.
