# Supplementary material for: Genetic Interaction Analysis Reveals that Cryptococcus neoformans Utilizes Multiple Acetyl-CoA-Generating Pathways during Infection
Source: mBio. 2022 Jun 29;13(4):e01279-22. doi: 10.1128/mbio.01279-22 (PMC9426453; doi:10.1128/mbio.01279-22)
Supplement: TABLE S1 [file mbio.01279-22-s0004.pdf]

No.	Protein (uniprot ID)	1	2	3	4	5	6
1	*Cn*Acs3 (J9VYY9)	-	-	-	-	-	-
2	*Sc*Psc60 (P38137)	22	-	-	-	-	-
3	*Cn*Acs (J9VFT1)	20	22	-	-	-	-
4	*Sc*Acs2 (P52910)	20	20	54	-	-	-
5	*Cn*Kbc (J9VT24)	15	18	26	25	-	-
6	*Sl*Kbc (D6EQU8)	15	18	26	26	38	-
