# Supplementary figures and images for: Genetic Interaction Analysis Reveals that Cryptococcus neoformans Utilizes Multiple Acetyl-CoA-Generating Pathways during Infection
Source: mBio. 2022 Jun 29;13(4):e01279-22. doi: 10.1128/mbio.01279-22 (PMC9426453; doi:10.1128/mbio.01279-22)

**A** MW (kDa)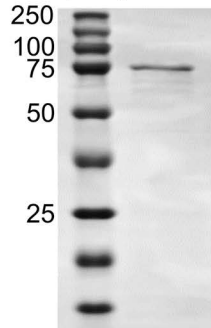**B**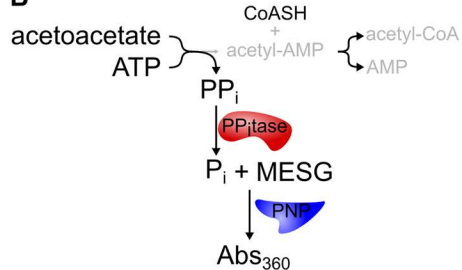**C**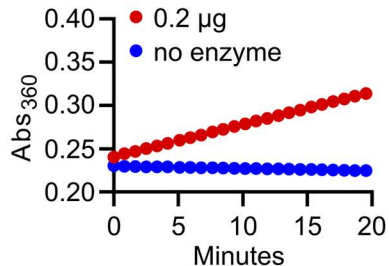**D**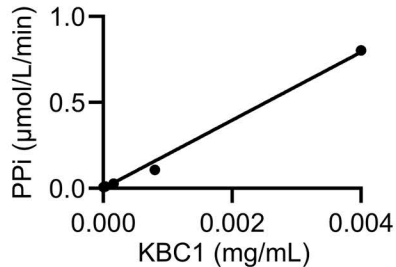**E**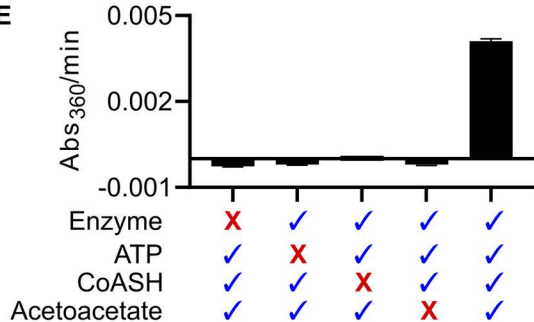**F**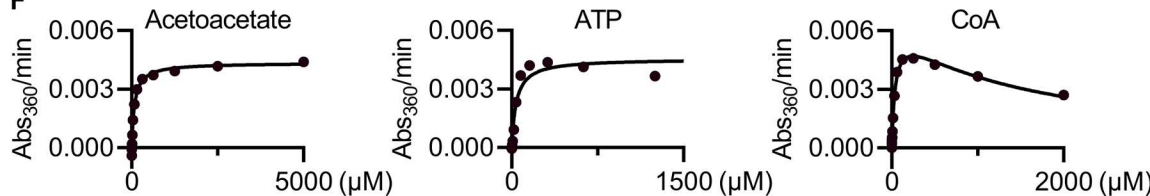

Supplement: FIG S1 [file mbio.01279-22-s0001.pdf]

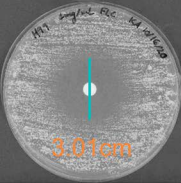

H99

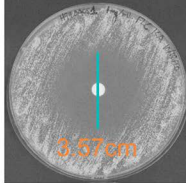

*acs1*Δ

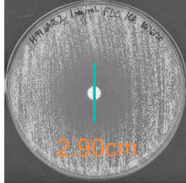

*kbc1*Δ

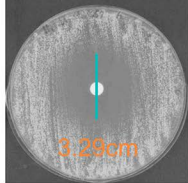

*acs1*Δ*kbc1*Δ

Supplement: FIG S2 [file mbio.01279-22-s0002.pdf]

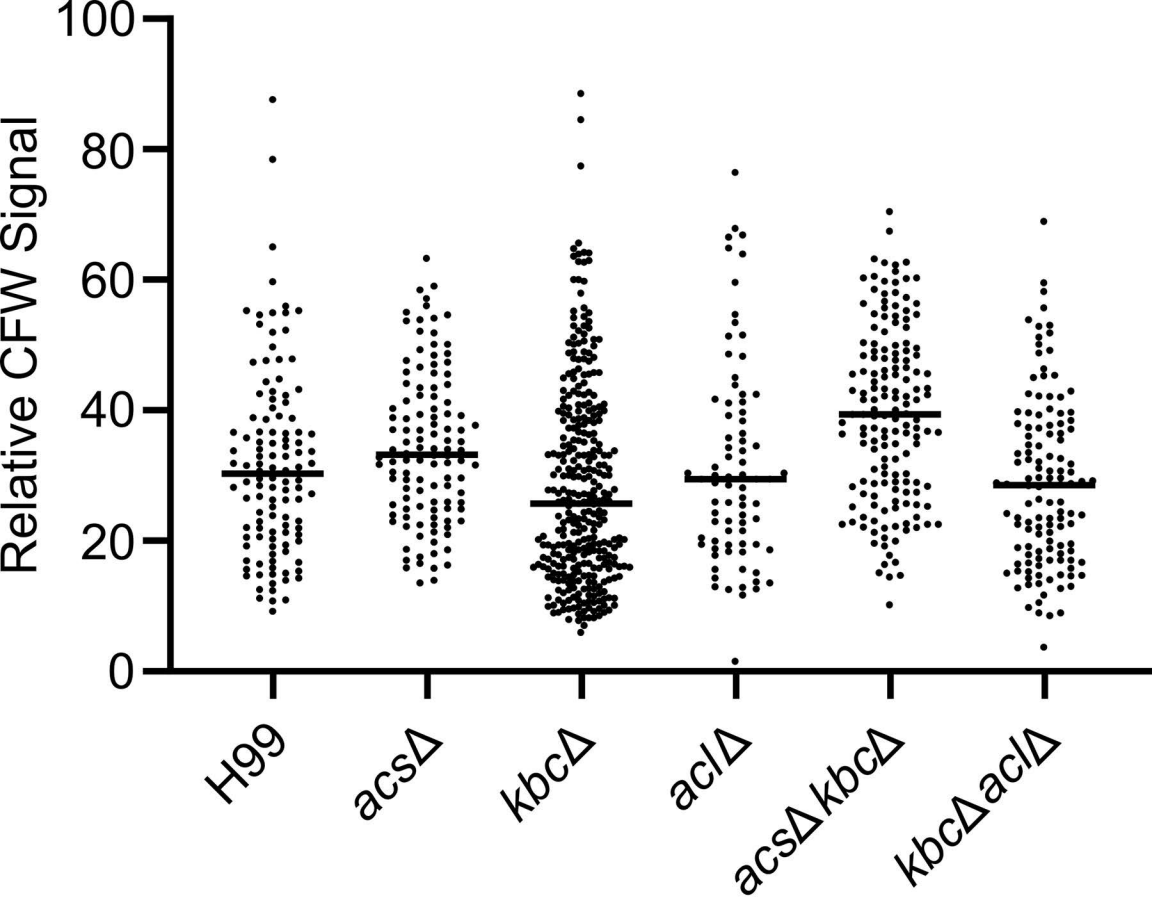

Supplement: FIG S3 [file mbio.01279-22-s0003.pdf]
