# Supplementary material for: Genetic Interaction Analysis Reveals that Cryptococcus neoformans Utilizes Multiple Acetyl-CoA-Generating Pathways during Infection
Source: mBio. 2022 Jun 29;13(4):e01279-22. doi: 10.1128/mbio.01279-22 (PMC9426453; doi:10.1128/mbio.01279-22)
Supplement: TABLE S2 [file mbio.01279-22-s0005.pdf]

Media with noted carbon source	H99	*acs1Δ*	*kbc1Δ*	*acl1Δ*	*acs1Δ kbc1Δ*	*kbc1Δ acl1Δ*
Minimal Media						
β-hydroxybutyrate	-	-	-	ND	-	ND
RPMI-MOPS						
0.2% glucose	++	++	++	+	++	+
0.2% glucose +10% FBS	++	++	++	+	++	+
Yeast Nitrogen Base						
2% acetate	++	-	++	++	-	++
2% acetoacetate*	-	-	-	ND	-	ND
2% ethanol	++	-	++	ND	-	ND
2% glucose	++	++	++	++	++	++
2% glycerol	++	++	++	ND	++	ND
2% leucine	+	-	+	+	-	+
Yeast Peptone						
2% acetate	++	-	++	ND	-	ND
2% dextrose	++	++	++	++	++	++
2% ethanol	++	++	++	ND	++	ND
2% glycerol	++	++	++	ND	++	ND

*acetoacetate was top spread
